# A conceptual model for chronic hepatitis B and content validity of the Hepatitis B Quality of Life (HBQOL) instrument

**DOI:** 10.1186/s41687-023-00675-8

**Published:** 2024-03-04

**Authors:** Jane Abbott, Natalie V. J. Aldhouse, Helen Kitchen, Hannah C. Pegram, Fiona Brown, Malcolm Macartney, Angelina Villasis-Keever, Urbano Sbarigia, Tetsuro Ito, Eric K. H. Chan, Patrick T. Kennedy

**Affiliations:** 1 Barts and The London School of Medicine and Dentistry, London, UK; 2 Clarivate, Manchester, UK; 3 Janssen Research & Development, High Wycombe, UK; 4 Janssen Global Services, LLC, Raritan, NJ USA; 5 Janssen Pharmaceuticals, Beerse, Belgium; 6 Janssen Health Economics & Market Access EMEA, High Wycombe, UK; 7 Barts and The London School of Medicine and Dentistry, Queen Mary University of London, Newark Street, London, E1 2AT UK

**Keywords:** Chronic hepatitis B, Clinical outcome assessment, Health-related quality of life, Patient-reported outcome, Quality of life

## Abstract

**Background:**

There is increased emphasis on incorporating patient perspectives and patient-relevant endpoints in drug development. We developed a conceptual model of the impact of chronic hepatitis B (CHB) on patients’ lives and evaluated the content validity of the Hepatitis B Quality of Life (HBQOL) instrument, a patient-reported outcome tool for use in clinical studies, as a patient-relevant endpoint to measure health-related quality of life in patients with CHB.

**Methods:**

A literature review of qualitative studies of patient experience with CHB and concept elicitation telephone interviews with patients with CHB in the United Kingdom were used to develop a conceptual model of the experience and impact of living with CHB. The content validity of the HBQOL was evaluated using cognitive debriefing techniques.

**Results:**

The qualitative literature review (N = 43 publications) showed that patients with CHB experience emotional/psychological impacts. During concept elicitation interviews (N = 24), fatigue was the most commonly reported symptom, and most participants were worried/anxious about virus transmission and disease progression/death. A conceptual model of patients’ experiences with CHB was developed. The conceptual relevance and comprehensibility of the HBQOL were supported, though limitations, including the lack of a self-stigma item and recall period, were noted for future improvement.

**Conclusions:**

The conceptual model shows that patients with CHB experience emotional/psychological impacts that affect their lifestyles, relationships, and work/schooling. The cognitive debriefing interviews support the content validity of the HBQOL as a conceptually relevant patient-reported outcome measure of health-related quality of life.

**Supplementary Information:**

The online version contains supplementary material available at 10.1186/s41687-023-00675-8.

## Background

Hepatitis B virus (HBV) is a major global health issue. Of ~296 million people worldwide living with chronic hepatitis B (CHB) [1], ~20–30% will develop cirrhosis, liver failure, or hepatocellular carcinoma [2]. CHB also has a negative impact on people’s health-related quality of life (HRQoL), including social and emotional functioning [3–5]. In addition to improving survival, a major goal of therapy for patients with CHB is to improve HRQoL [2, 6].

The US Food and Drug Administration established the patient-focused drug development (PFDD) initiative [7, 8] to stimulate the systematic study and incorporation of patients’ experiences and needs into drug development and evaluation. Despite this, the assessment of CHB treatments continues to focus on clinical and laboratory outcomes (e.g., HBV DNA suppression, decline in hepatitis B surface antigen [HBsAg], liver biopsy results) while neglecting HRQoL and other patient-focused outcomes (e.g., disease-associated symptoms, treatment experience, impact of living with CHB on HRQoL). Evaluating these patient-focused outcomes is essential to informing patient-centered measurement strategies [9–14] and is key to bringing meaningful, effective, and deliverable results to patients with CHB worldwide [4].

One way to capture patient perspectives is through the use of patient-reported outcomes (PROs), defined as any report of health status coming directly from patients without anyone else’s interpretation [15]. PROs play an important role in PFDD and are commonly used to assess the psychosocial and HRQoL outcomes that matter most to patients [16]. They are being considered as emerging biomarkers in the field of chronic liver disease [17].

Recommendations for the design of clinical trials of therapies for CHB focus on the assessment of functional cure endpoints, defined as sustained loss of HBsAg with or without HBsAg seroconversion and undetectable HBV DNA in serum [18]. Such endpoints do not assess the patient experience or changes in HRQoL while on chronic treatment. When we began this study, the Hepatitis B Quality of Life (HBQOL) questionnaire was the only disease-specific PRO measure designed to assess HRQoL in patients with CHB. The HBQOL questionnaire is a 31-item measure organized into 6 domains (psychological well-being, anticipation anxiety, vitality, stigma, transmission, and vulnerability) [3]. More research is needed to investigate the degree to which the HBQOL items and domains are comprehensive and representative of the HRQoL concepts that are important to patients with CHB. Qualitative interviews, the recommended methodology for establishing the content validity of a PRO measure intended to support endpoints in clinical trials, are key to demonstrating the measure’s overall score validity, as outlined in regulatory guidance documents and best practice recommendations [15, 19–22].

The first objective of this study was to build a conceptual model of the experiences of people living with CHB. The second objective was to evaluate the content validity of the HBQOL instrument.

## Methods

### Study design and patients

A conceptual model captures the experiences that people living with a condition find most important, and the development of a conceptual model is an essential step when selecting PROs for inclusion in clinical studies [23]. The current study developed such a conceptual model by conducting a literature review of qualitative studies of CHB and interviews of people living with CHB, encompassing pre-diagnosis, treatment, and living with a chronic condition [24]. Interviews with patients also served to evaluate of the content validity of the HBQOL, which was first developed in 2005. Though some evidence of content validity for the HBQOL was reported at the time of the measure’s development, the field of clinical outcome assessment has since advanced [15]. The current study expanded on available evidence regarding the content validity of the HBQOL in a number of ways: (1) we fully explored and documented, from the patient perspective, the patient experience of CHB in order to confirm the relevance and comprehensiveness of the assessed concepts, (2) we assessed and documented whether the HBQOL’s wording and response options were comprehensible by patients, and (3) we explored whether potential recall periods were appropriate for addition to the measure in order for it to be usable to monitor longitudinal changes in patient status.

### Literature review

A targeted literature review of qualitative studies in patients with CHB was conducted by searching the Embase, Medline, and PsycINFO literature databases using search strings that combined terms such as chronic, qualitative, interview, and hepatitis B (Table S1). Manual searches of relevant conference proceedings were also performed.

Qualitative studies in patients with CHB, published in English from 2000 to 2020 as journal articles, conference abstracts, and gray reports, were selected for inclusion. Studies reporting on the lived experience of patients from the perspective of non-patients (e.g., clinician, family, friend, caregiver) were excluded. The Critical Appraisal Skills Programme checklist [25] was used to assess the quality of the identified publications. The included articles were analyzed using a thematic synthesis approach (facilitated by ATLAS.ti v7 software) to identify symptom/side effect and impact concepts.

### Selection of interview participants

Interviews were conducted to (1) supplement the literature review as part of conceptual model development (concept elicitation) and (2) evaluate the content validity of the HBQOL (cognitive debrief). Participants were recruited from the viral hepatitis clinics at Barts Health NHS Trust, London, UK. Eligible participants were identified by recruiting physicians following review of patient medical records at their practice site. According to published evidence [26] and experience, ~12 interviews should be sufficient to achieve concept saturation in a relatively homogenous sample.

The study was open to adults (aged ≥ 18 years) with a diagnosis of CHB of any genotype and adequate knowledge of the written and spoken English language. Additional key inclusion and exclusion criteria are included in the supplementary materials. This study was reviewed by the local ethics committee/institutional review board and was performed in accordance with the ethical principles of the Declaration of Helsinki. All study participants provided written informed consent.

### Interview process

Interviews were conducted by phone by a Barts Health NHS Trust–affiliated specialist researcher. During concept elicitation, the interviewer used open-ended questions to explore multiple aspects of participants’ experience of living with CHB. During cognitive debriefing, the interviewer asked participants to complete the HBQOL questionnaire [3] using a “think aloud” technique whereby they spoke their thoughts as they read items and selected an answer. During the HBQOL conceptual framework section, the interviewer asked participants to rank the domains of the HBQOL according to which experience was most important to them.

### Interview analysis

Assisted by ATLAS.ti qualitative data analysis software, Clarivate analyzed the concept elicitation interview transcripts using methods based in thematic analysis [27]. Concept elicitation data were then reviewed to assess conceptual saturation, defined as the point at which no new concept-relevant information was identified related to signs, symptoms, or impacts. The cognitive debriefing interview transcripts were analyzed using framework coding [28] whereby a pre-defined code list was applied to identify the relevance and appropriateness of the measurement concept, instructions, item wording, response scale/options, and recall period.

## Results

### Qualitative literature review

**Fig. 1:**
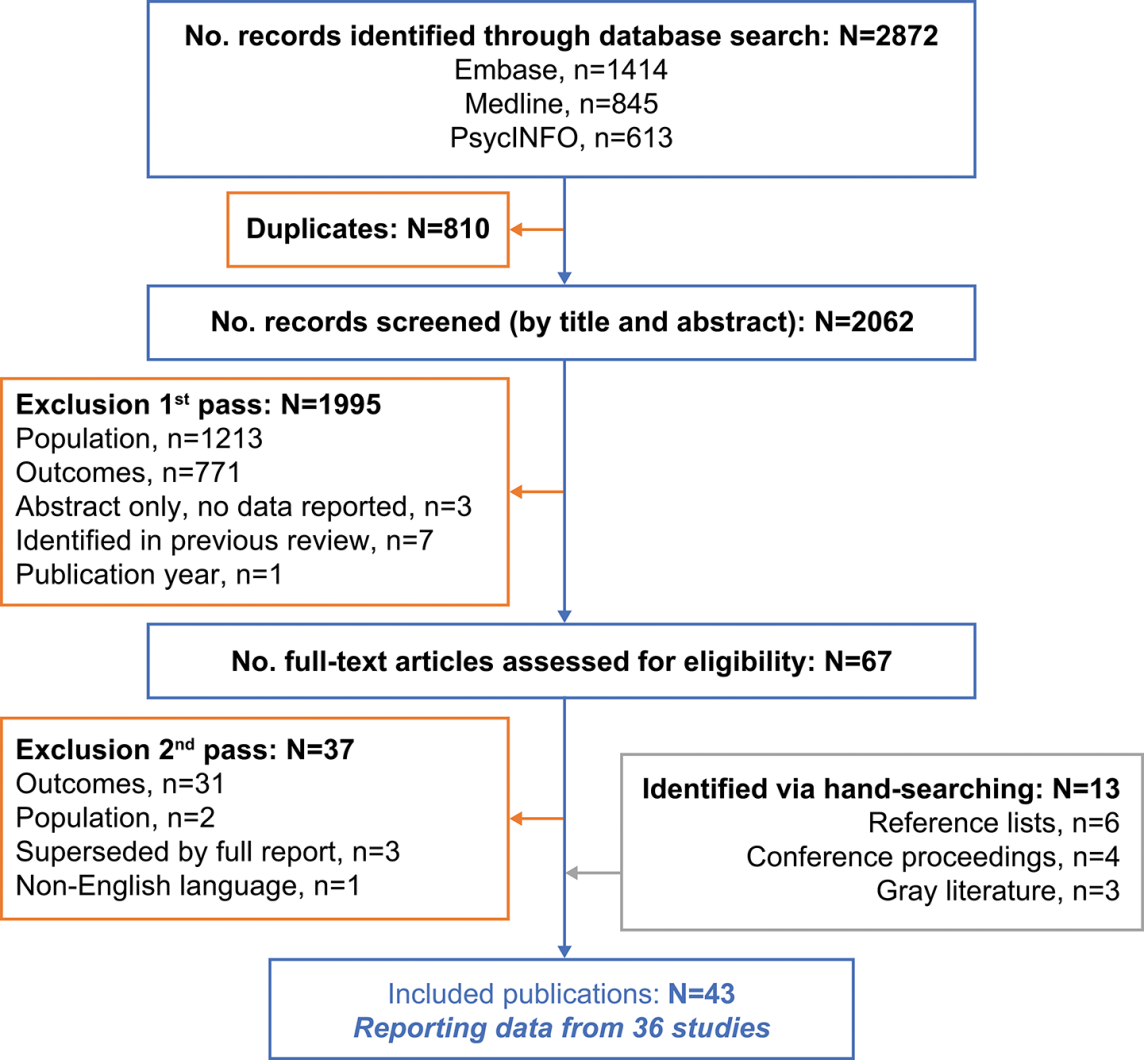
Selection of studies for qualitative literature review

Data examining the experiences of people living with CHB from 36 qualitative studies, reported in 43 publications that met the inclusion criteria, were reviewed (Fig. 1). The publication details are summarized in Table S2. The 43 publications reviewed reported findings from 36 studies worldwide; 42 of 43 publications reported the number of participants (mostly [30/42] published in peer-reviewed journals) and included 3808 participants (range of 4–1838).

The qualitative literature review (citations listed in Table S2) revealed that although many people with CHB were asymptomatic, symptomatic individuals experienced numerous symptoms, including flu-like/gastrointestinal symptoms, fatigue, weight loss, jaundice, and pain. Throughout life, CHB significantly impacted participants’ emotional and psychological well-being; existing relationships with partners, family, and friends; and development of new relationships. People with CHB experienced social stigma and self-stigma, and work and school were affected. The impact of CHB on work and lifestyle limitations was associated either with the physical symptoms/side effects of CHB and its treatment or with (often considerable) social stigma. Coping strategies included dietary/lifestyle changes and emotional management.

### Characteristics of interview participants

Twenty-four adults with CHB living in the United Kingdom participated in qualitative interviews. The mean age of participants was 39 years, with a mean (range) time since diagnosis of 11 (2–30) years (Table 1). Most participants were Asian/Asian British (42%) or Black/African/Caribbean/Black British (33%).

**Table 1:** Baseline demographics of interview participants

Characteristics	(N = 24)
**Age, years, mean (range)**	39 (27–56)
**Gender, n (%)**	
Male	13 (54)
Female	11 (46)
**Employment status, n (%)**	
Paid work	21 (87)
Unpaid work	2 (13)
**Highest qualification, n (%)**	
Master’s degree or postgraduate certificate	7 (29)
Bachelor’s degree	10 (42)
AS level/A level/International Baccalaureate	2 (8)
BTEC diploma	1 (4)
O level/CSE/GCSE	4 (17)
**Ethnicity, n (%)**	
Asian/Asian British	10 (42)
Black/African/Caribbean/Black British	8 (33)
White	5 (21)
Romanian	1 (4)
**English language, n (%)**	
Not first language	15 (63)
First language	9 (38)
**Years since diagnosis, mean (range)**	11 (2–30)
**CHB treatment history, n (%)**	
Currently on treatment	18 (75)
Treatment naive	5 (21)
Previously treated	1 (4)

*AS* Advanced Subsidiary, *BTEC* Business and Technology Education Council, *CHB* chronic hepatitis B, *CSE* Certificate of Secondary Education, *GCSE* General Certificate of Secondary Education

### Concept elicitation

#### Patient-reported symptoms of CHB

Key findings from the concept elicitation interviews are listed in Tables 2, 3, 4, 5, 6, 7, 8, S3, and S4. When asked how CHB had affected them physically, 13 of 24 participants said that they had not generally experienced physical symptoms. When asked about specific physical symptoms, fatigue was the most commonly reported (n = 16). Participants described fatigue in terms of feeling tired/sleepy (n = 14), lack of energy (n = 4), and weakness (n = 3; Table 2). As stated by one patient, “Since I got the virus, I don’t have enough strength and I get really tired. Every time I want to lean on my hand or and I can’t do it because there’s not enough strength and it start hurting to bone. There’s no strength in my body and I’m putting too much pressure on me. Yeah. I get really tired so quickly and that’s so much.” Other symptoms frequently described by participants included abdominal pain (n = 6), cognitive issues (n = 4), and weight changes (n = 3).

**Table 2:** Symptom concept elicitation—summary of responses and example quotes

Symptom	No. reporting (N = 24)	Example quotes
Fatigue: tired/sleepy	14	“Since I got the virus, I don’t have enough strength and I get really tired. Every time I want to lean on my hand or and I can’t do it because there’s not enough strength and it start hurting to bone. There’s no strength in my body and I’m putting too much pressure on me. Yeah. I get really tired so quickly and that’s so much.” “It’s just I feel– I generally sometimes just feel tired. Like if I look at the computer screen for too long or I’m trying to work, my concentration goes down and I just feel like I want to sleep. But as I said, I’m not sure if it’s just because of the condition, or it’s just me.” “There are times when I feel very tired, extremely tired, and for some unknown reason. And I think I’m not too sure if that has anything to do with what I got apart from me working hard a lot.”
Pain: abdominal	6	“I’ve got pains in my– on the right-hand side just below the rib cage where the liver is.” “I do get pains in my liver, like shooting pains in my liver.”
Fatigue: lack of energy	4	“The main way I think is because I’m not feeling like last ten years, and now it’s coming to ten years, feeling I’m not as energetic like before.”
Fatigue: weakness	3	“It definitely makes you– physically makes you weak.”
Cognitive: foggy/difficulty concentrating	4	“In terms of work, it does– you can lose concentration, etc., but as I said, I’m not sure if that’s just one of the side effects of the medication and the condition.”
Weight change	3	“I would say, first six months, I found it very, very difficult. I lost lot of weight. I lost a significant of weight and there’s no strength in my body, and there was no I couldn’t eat. I could not even eat.”
Dizziness/feel faint	2	“I was palpitating. Most times, I have to lay down on my bed most times because, at that period in time, it affected my liver to a very good extent.”
Gastrointestinal: heartburn/indigestion	2	“It appears to take longer for me to digest stuff, so if I eat around a significant time […] when I was, I mean, years ago, I will be uncomfortable.”
Poor vision	2	“I think my eyesight is getting worse now because now the things I can– before more blurry, less blurry are becoming more blurry. So that’s something that’s really going to affect you more physically.”

#### Impact of CHB

Participants were asked to describe the initial impacts of CHB diagnosis on their lives and how these feelings might have changed over time (Table S3). Commonly reported experiences included feeling shocked/overwhelmed/devastated (n = 6) and confusion (n = 5).

Then, participants were asked to describe the impacts of CHB on their lives. Example quotes are provided from these interviews related to emotional/psychological well-being (Table 3), social functioning (Table 4), relationships (Table 5), activities of daily living (ADL; Table 6), and work/school (Table 7). In terms of the impact of CHB on their emotional/psychological well-being, most participants were worried or anxious about transmitting HBV (n = 18) and about disease progression/death (n = 17). For instance, one patient stated, “I’m, yeah, constantly worried about what comes next. Am I going to develop something with my liver earlier than expected or soon?” Other feelings expressed included being depressed/down/upset (n = 6), anxious/worried about disclosing their diagnosis/being exposed (n = 5), being annoyed/frustrated/irritable/angry (n = 4), stress (n = 3), and low self-esteem/confidence (n = 3).

**Table 3:** Impact on emotional/psychological well-being concept elicitation—summary of responses and example quotes

Impact on emotional/psychological well-being	No. reporting (N = 24)	Example quotes
Anxious/worry about transmitting CHB to others	18	“I became cautious when I’m around people. I feel like I don’t want to be responsible for infecting somebody because of whatever comes from my body is going near them or whatever it takes. And it makes me stay away from people sometimes, especially my friends.” “Because I’m not on medication, I do get worried when I get a cut or any bleeding or anything, because I don’t want to kind of infect any others and I don’t really want to tell people I have it, kind of thing.” “I’m thinking of, like, families. I don’t want to pass it on to my children. Really, really focused on avoiding that, an absolute possible. So that is quite emotional for me, because I’m thinking. I’m not just affecting myself, I’m affecting my family, my daughter, perhaps. The likelihood they’re going to have it and they’ll going to deal with the same thing, having from birth. Yeah, so that’s quite hard to kind of swallow, really. Yeah.”
Anxious/worry about disease progression/death	17	“I’m, yeah, constantly worried about what comes next. Am I going to develop something with my liver earlier than expected or soon?” “I don’t know where that leads me in the future, because I know my uncle who lived in [retracted], he had Hep B and then it damaged his liver in cancer. So they caught that all too late, and he’s passed away about three years ago. So I’m thinking would that affect my future almost?” “Very nervous. Very nervous and very conscious. And one term I would say, scared, because I remember the first thing the doctor would tell me that I still think about every day is that, soon as she find out and she said, “Oh, that might lead to cancer,” before even telling me, explaining to me. And as soon as she said that, since that day, I’m still thinking, “Oh, I still got the virus. I might get cancer.” And then also stuff. Very conscious.”
Depressed/feeling down/upset	6	“Emotionally, it can be depressing. Yeah, it can be very, very depressing because you just– you withdraw yourself. You feel like it– just because you’ve been happy, it just became a mental block in your livelihood.” “Last summer, I went through depression and anxiety. And I think the hepatitis B actually created big problem there. Yeah. I took professional advice and I’m much better now. But I guess my because I have a family. I got a wife and I got a little boy, and I just always back of my mind, it’s just like, “If things happen to me, I don’t what’s going to happen to my family and my wife?” Yeah. As I said beginning of my conversation, it stick to my brain and it doesn’t, but it does affect me emotionally.”
Anxious/worry about disclosing/being exposed	5	“I have to tell other people because I can’t hide it from work. I have to tell other people and then I’m worried who my boss might tell. I kind of feel like I’m going to be in a kind of bubble, whereby, people who are naïve about it or who doesn’t know anything about it, they might feel like it’s something that I might pass onto to them and then they would be careful in relating with me socially or engaging with me. It makes me kind of feel it makes me reject myself from the outside world a lot, actually, if I’m being honest. I have less friends right now. Not because they don’t want to talk to me or it’s because I reject bring myself a little bit because I don’t want to be forced to tell people what I’m living with or what I’m going through. I don’t want to be forced that, when they see me, if I’m taking my medication every morning, they ask me questions, then I don’t want to have any reason to lie for anyone. As such, I kind of move back from friends.”
Annoyed/frustrated/irritable/angry	4	“Frustrated. Very frustrated. Angry as well because we don’t know where it comes from. There’s a history of it in the family. My mother has it and she advanced to liver cancer. And my younger sister she has less severe condition than I. I don’t think she has chronic. I have two other siblings preferably okay. So it’s just that frustration not knowing where it came from and my mother doesn’t know either. I think the whole family went for a check in our teens and a diagnosis was picked up then but she– the parents didn’t do anything about it. So, neglectful and a bit angry that my– I moved onto developing chronic when it could have been prevented or contained.”
Stress	3	“I was having ups and downs about it, and it gave me a lot of stressed nights because I wasn’t too sure where I am and how I’m going to get through it.”
Impact on identity	3	“Actually, it makes me an extra cautious person because it makes me feel ashamed, to be honest. And ashamed in the fact that I think it comes down to my culture and my history because I am this kind of person. I’m a born [redacted] and, in my family, we’re extremely religious. And as such, I query myself how and where I could have come across. I understand it could be through sexual intercourse and those. I am one of I am extremely careful when it comes to that. Another is maybe blood transfusion, which I never had one and I never give blood before or anything, so I know that. And I never inject myself with anything or such. It made me worried of where might have contracted it over the course of time.”
Low self-esteem/confidence	3	Interviewer: What do you think your life would be like if you didn’t have Hepatitis B? “Just I’d feel freer. I’d be much more socially confident, especially in terms of partners. I’ve kind of resigned to the fact that I won’t find anybody. I don’t try either but yeah definitely I’d feel more confidence, more sociable as well.”
Feel guilty about the impact on others	1	Interviewer: Can you tell me how it’s affected you being around others? “I feel guilty because they don’t know, but I’m also scared of telling them because of the reception that I may get back from it.”
Suicidal thoughts	1	“It kind of made me– to be honest, I just didn’t want to be here, like, on this planet at all. I just felt so low to myself and– yeah…It just made me want to give up on life in general.”

*CHB* chronic hepatitis B

**Table 4:** Impact on social functioning concept elicitation—summary of responses and example quotes

Impact on social functioning	No. reporting (N = 24)	Example quotes
Precautions to avoid transmission	13	“If you got injured the moment you start– blood start coming out, you’ve got to let everybody know what you have. So we keep on with difficult to do to try and avoid injuries and everything. So you become more extra careful. So it became a burden on your head for, like, I don’t want to do this, I don’t want to do that, because some of the things you get, you might get injured, you might start whatever it is, the sweat and all it, bits and rocks, and they feel like close friends, yes, sometimes you don’t want them to know your things, but you’ve just got to let people know what you have to protect them and protect yourself, whatever it is.” “It does affect me, especially, as I said to you, when you’re socializing, I can’t socialize with my colleagues as much as I want because, obviously, I have hepatitis B and I still have the virus in me. I’m always conscious about not being too close or not sharing the food or not being able to do as everyone else can do, you understand? Only go out, especially with the colleagues and stuff, having a drink and stuff, I can’t do that as much as I wanted to. That’s the social side of it.”
Reduced social life/feel lonely	6	Interviewer: what do you think your life would be like if you didn’t have hepatitis? Would it be different? “It would be different, yes. I believe so. I think I will much more, much more happy. I wouldn’t have hide in my own shells and not wanting to engage with others. I think I would my life would be ten times better than where I am right now. But I think, day-by-day, as days goes by, I feel better now, but I still feel like I’m isolating myself.” “You cannot just go out and make friends because people won’t all understand, and maybe they don’t want to be near or risk anything.”
Unable to reach personal/life goals– career goals	5	“I think I was just looking for the child minding or the same stuff. Like, now I’ve just dropped that idea because the other person, they have to do the vaccine or the stuff that makes me scared.” Interviewer: Okay. So that stops you looking at that line of work. “Yes.”
Unable to reach personal/life goals– family life	2	“Emotionally it is quite worrying because I might wait too late to have what I want in life and what I can’t, so it’s just like a constant fear of I have children and stuff like that.”
Unable to reach personal/life goals– unable to donate blood	1	“I’ve been concerned that because of that I will not be able to donate blood or anything like that.”

**Table 5:** Impact on relationships concept elicitation—summary of responses and example quotes

Impact on relationships	No. reporting (N = 24)	Example quotes
Impact on family role	6	“Because I had it when I was really young, I didn’t really know kind of like what it mean, kind of like the implications, like exactly how it– I mean, but, when I’m older, it’s kind of like, more or less get worried, kind of like when I met my partner, kind of like being open about it, and then planning the future, like I want to have children, how it may affect me. So I think there’s more– I think the older I am, then I think– there are some aspects that affect me, yeah.” “Physically, it affects because innovation, especially cooking, you need to do a lot of cooking sometimes and you have like three, four, five hours in the kitchen and this stuff and it makes you tired, standing for long times, because before I never feel tired when I do the whole family would be patient. But now I feel very tired, even when I cook for two or if I have to machines and I cook half the time.”
Difficulty dating/starting relationships	5	“So fifteen years now and I’ve not had a partner since then so obviously it’s just that anticipation not trying to find a partner, not putting myself out there to meet people because of it. Purely because of that reason.”
Impact on intimacy and sexual relationships	4	“I would have to confess that it does impact my relationships. And that in hindsight, actually, because at the beginning I thought, okay, I would just live with it. I thought about the doctors, the [redacted] doctors who explained clearly that you can live with it. But after two or three years, I do realize that it– I do– I’m letting relationships evolve into sexual relationships, for instance, much less than before.”
Relationship strain/fear relationship may end	2	“But it’s actually– it’s a bit more difficult for my partner because he doesn’t really have the same level of assurance that I’m not a threat to other people that I do. I’ve lived with it. So, I can judge if it’s dangerous or not. Where for him, he suffers with OCD a little bit. And so, for him, everything is a threat. Even family members and potentially sharing meals. Things that we know that we’ve asked the doctor. They’re not things that will harm other people. But for him, he doesn’t know where the boundaries are and what the guidelines are. So, it’s difficult for him. And, then, that makes it difficult for me.”

**Table 6:** Impact on lifestyle/ADL concept elicitation—summary of responses and example quotes

Impact on lifestyle/ADL	No. reporting (N = 24)	Example quotes
Limitations/burden of sickness/appointments/treatments	7	“I wouldn’t have to remember to take medicine every day. So there would be so much easier for me, and I wouldn’t have to carry pills around whenever I travel and make sure I don’t lose the pills. Yeah, I wouldn’t have to spend so much time in hospitals.” “Well, my experience it’s not like I guess having a normal life. Normal, I mean, not having it, you know, I found out when my wife was pregnant and so It’s not like I have known my whole life. That puts everything into perspective and that sort of puts some steps obviously that’s your life. And you can’t do things that you think is normal for other people to do everything, and I have to stop and consider everything–is it okay if I do this, or is it okay if I do that.”
Can’t eat/drink what I want	6	“I think about what I’m eating. Not to say I’m modifying it, but I’m at least wondering how it– how it impacts my liver, for instance.” “It used to be a bit more difficult at the beginning obviously because I was very young, and I wanted to be drinking and eating whatever fast foods I wanted, things like that, and I still do all of those things, but very, very limited. And yes, when I do drink I do think about maybe I shouldn’t drink more than two glasses or three max, you know, look after myself and my liver ultimately.”
Physical/exercise limitations	4	“Playing football, you feel like, no, I might get injured and things like that. And I tell people, there’s quite a few people at a football game. Not everybody’s playing so I guess watch it.”
Increased resting/napping	3	“My daily life, I’m a [redacted]. I work in the [redacted], so you can imagine I need to be pretty much active every day. That comes in is the energy, so I don’t really get sometimes […] if I’m doing a lesson for two or three lessons, no break, I get very, very tired. Very, very tired. If I sit down, I sadly just fall asleep just for 10 min because there’s not enough energy in my body.”
Sleeping difficulties	2	“And then I feel as well and I couldn’t sleep sometimes properly. At night, sometimes I wake too long.”

*ADL* activities of daily living

**Table 7:** Impact on work/school concept elicitation—summary of responses and example quotes

Impact on work/school	No. reporting (N = 24)	Example quotes
Time off to attend appointments	6	“I’m able to take a day or half a day of every week for when I need to make to [redacted] hospital. And yeah, there’s never been a question.” “In terms of work, I think I feel like I have to do more appointments, you know, working with [retracted] and getting, like, monitored all the time. So that doesn’t just go with my work, getting time off for that, and explaining it to my, you know, [redacted].”
Ability to work/study	5	“It affects my daily life because, to start with, initially, I couldn’t walk and that is something that it was something very major for me at the beginning. I couldn’t go to work for many months.”
Difficult to get a job/be accepted at school	2	“To be honest, back home I didn’t work. I didn’t have too many jobs. Maybe back home was a little bit hard, and I struggled. But here, no.” Interviewer: And can you tell me a bit more about back home? What are the problems there? “To be honest, there, if you work in hospitality service– like waitress and this kind of stuff, or in the kitchen– they do some blood tests a lot. And with a hepatitis B– I don’t know how shall I tell you? They don’t like it too much. I don’t know. Maybe that mentality of the communist period of time. I don’t know, to be honest. Even in a hospital if you find a job, it’s the same. You need to be very healthy and stuff like that.” Interviewer: Okay. So, very different here, it sounds like, in that way? “Yes. When I came to [redacted], I find it very different way. At the beginning, I was worried because all– I said, okay. But, then, they said, no. You can do this job. You can do this one. Don’t worry. You are fine.”
Fear of losing job	1	“Before you can be a registered manager to own a care agency, there was a particular […] form that I had to send to Care Quality Commission and that piece form actually asks specifically if you have hepatitis B. When I saw it, before I fill up my form it took me almost two weeks before I could complete that little piece. What should I say? And then, at that point in time, because I was worried that they won’t register me to be the without being register to be a registered manager, I can’t own a care agency. I then begin to search for options of looking to employ somebody for that position. And then my biggest worry then becomes not having enough funds to do so, so I don’t know how to move on those to do it. And at some point it was like, ‘You know what? What will be will be.’ I should just tell the truth, right? Because I’m not willing to lie about that because I don’t know what would come up after. Then pen it down. I just say, ‘Yeah. I do have hepatitis B.’ And then I attached the notes that how I’m managing, I clear the space for myself, I’m going to be having my own office, and things like that. And then yeah, they didn’t even query, so I got register as registered manager. That went a very long way for me of that was a big concern for me in reference to my job at that point in time.”

Having to take precautions to avoid transmission to others (n = 13) was the most reported impact of CHB on participants’ social functioning (Table 4). One participant said, “I’m always conscious about not being too close or not sharing the food or not being able to do as everyone else can do…” and another one said, “So it became a burden on your head for, like, I don’t want to do this, I don’t want to do that…” Other frequently mentioned impacts on social functioning were reduced social life and feeling lonely (n = 6) and being unable to reach personal/life/career goals (n = 5).

Impacts on relationships included impact on family role (n = 6), difficulty dating/starting relationships (n = 5), and impact on intimacy and sexual relationships (n = 4; Table 5).

Regarding the impacts of CHB on lifestyle and ADL, many participants discussed limitations such as the burden of sickness, and appointments/treatments (n = 7) and not being able to eat and drink what they wanted (n = 6; Table 6). Some participants spoke of the need to regularly take medication and attend frequent medical appointments; others discussed the impact of having an incurable lifelong illness governing their choices. Participants who talked about the inability to eat or drink what they wanted usually mentioned alcohol consumption. Other impacts on ADL included physical/exercise limitations, increased napping/resting, and sleeping difficulties.

The most frequently mentioned impacts of CHB on work/school were related to the need to take time off to attend appointments (n = 6) and the ability to work or study (n = 5; Table 7). Participants talked about the negative effect of incapacitation from increasing viral load and the impact of symptoms (e.g., fatigue, abdominal pain) on performance. Other impacts of CHB on working life included difficulty obtaining employment and fear of losing work.

#### Coping with CHB

In another portion of the concept elicitation interviews, participants were asked to talk about how they were coping with CHB (Table S4). The most frequently described coping techniques reported by participants were acceptance (n = 14), engaging with regular medical care (n = 10), support from family and friends (n = 7), and improved disease understanding (n = 7). Other coping strategies included taking general health precautions, reducing alcohol intake, seeking emotional support from health care professionals, modifying diet and exercise, emotional avoidance, and ensuring sufficient rest.

#### CHB-related stigma

The concept elicitation interviewer also asked participants if they had ever felt stigmatized, and/or experienced prejudice or discrimination because of CHB. As summarized in Table 8, 3 categories of stigmatization (i.e., social stigma, self-stigma, institutional/structured stigma) based on a World Health Organization report [29] and published literature were used [30, 31].

**Table 8:** Experience of CHB-related stigma concept elicitation—summary of responses and example quotes

CHB-related stigma	No. reporting (N = 24)	Example quotes
Self-stigma: concealment	14	“None of my friends really know, none of them. If my partner did– my partner now, he knows, but other than that I don’t tell any of my friends.” “I’m concerned that if– the disease carries a stigma and people don’t really understand it, then they might think that it can be spread. So I don’t tell everyone that I have it.” “Well, I keep it secret. I don’t– there’s only a handful of people who know other than my family. My employer doesn’t know.”
Self-stigma	7	“I would say, and if I feel stigmatized, it’s probably more me thinking about it than someone verbally insults me or people walking away because they think I’ve got something viral.” “I haven’t felt stigmatized by anyone else, but I just, you know, when you think about it yourself then obviously you feel I’ve got this condition, and then you just feel bad.”
Self-stigma: expect avoidance from others	7	“My major worry is really rejection. And I think a lot of people have been naïve or have no idea what it’s all about. And because of that, I’m feeling like I don’t want to be the guy who needs to train people based on what I’ve got. At the initial stage, to convince people to see me as normal and that I’m not going to just pass any disease or anything to them at any time.” “You cannot just go out and make friends because people won’t all understand, and maybe they don’t want to be near or risk anything.”
Social stigma: misconceptions from others	7	“I got publicly humiliated in the workplace, and it made me– a lot of people stopped wanting to work with me because they thought they could just catch it by working with me, just brushing past me and stuff like that.” “How does it make me feel? There’s a thing where I feel like I have to explain myself if it ever comes up to people, and that’s always a really awkward conversation because, again, people immediately think it’s from a, you know, STI thing, that I’ve obtained. So that’s quite embarrassing, if that makes sense.”
Social stigma: judgment/talked about by others	5	“It’s sort of been a roller coaster of mixed emotions. When I did speak out about it, it wasn’t the reception I was expecting, so I kind of got singled out and pretty humiliated by the company I used to work for, and family members from my ex’s family.” “Okay. And have you ever felt judged by others or treated differently because of it?” “Yes, a little bit. There were a couple of times in my life when I have told my partner that, “Oh, look, I have this condition and blah blah blah. We just need to be careful for the time being, and then after on if we are more involved, you can have a vaccine.” But instantly it was, like, he didn’t talk to me, he got really upset, he went to bed, and then the next morning, once he had a bit of time to process the whole thing, he kind of accepted it and apologized and everything, and obviously it wasn’t my fault. But yeah, I just felt a bit weird.”
Self-stigma: avoidance/withdrawal from social situations with others	4	“I know for sure, deep down inside me, that I decided not to have too many friends because I don’t want to have to answer what is my situation to nobody. I kind of reject myself from the outside world. I do my own activities mostly with my partner. We do exercises and live a healthy life. But in terms of relating with other people, I kind of move back a lot in that sense.”
Self-stigma: feel embarrassed/guilty/ashamed	3	“Given the fact I read through the things that could have make it happen, that will have caused hep B, I began to feel slightly ashamed about myself, my condition. I go, “Where did I get this from?” It becomes a tricky situation for me at that time.”
Social stigma: avoidance by others/exclusion	3	Interviewer: What do you think your life would be like if you didn’t have hepatitis B? “People would have more trust and if they know that I am hep B positive, they wouldn’t avoid. They would like to be more friendly with me and wouldn’t be scared of me.”
Self-stigma: feel dirty/tainted/worthless/useless	1	“It makes me feel degraded because I feel like whatever happens, if I– if I’m not with my current– if I– every time I get a new partner, […], I mean, I’ve got to explain to them what I have. And that can put people off, having a relationship with you, because they’re going to feel like, okay, you are contagious. What have you been doing? What else have you got? It doesn’t matter what you tell them.”
Social stigma: others will not share items/food	1	“There’s few friends I’ve told. I thought one or two were– I thought they were– they were good, good, friends, but their perception changed. And when I told them that, go to their household, they came here, they were offered a tea or drink, they would not have it. And when I go to their house, I can see that– I go to their house a few times, I can’t make a cup of tea. Because before, I used to go out and make a cup of tea. I’d feel like, hey, I’ll help myself. But now, I can’t do that [INDISCERNIBLE] restrictions. One thing I realized, which is embarrassing and depressing, was every time I would go there, I was given one single cup, and that’s the cup I was to drink from. Looks like the other company, best I know, they can’t drink from that cup.”
Structural stigma: denied opportunities	1	Interviewer: And have you ever experienced prejudice because of your hepatitis B? “Not where I’m working now, but in my previous job in the [redacted] I had, I was just singled out all the time.” Interviewer: And in what kind of way if it’s not too upsetting to talk about? “Like, all the people who I thought were my friends who I worked with didn’t want to sit with me for lunch or didn’t want to work with me, and just I was overlooked for promotions and stuff.”

*CHB* chronic hepatitis B

Among participants, the most frequently reported experiences of stigmatization were self-stigma (n = 18; defined as self-induced, internalized negative belief that affects feelings and function any time an individual thinks negatively about themselves based on what other people might think or believe about them because of their health condition [29–31]), including concealment of CHB from others (n = 14), negative thoughts about themselves (n = 8), and expectation of social avoidance from others (n = 7). As described by one participant, “My major worry is really rejection. And I think a lot of people have been naïve or have no idea what it’s all about. And because of that, I’m feeling like I don’t want to be the guy who needs to train people based on what I’ve got. At the initial stage, to convince people to see me as normal and that I’m not going to just pass any disease or anything to them at any time.” Among the participants who described experiences of social stigmatization (n = 9), the most frequently mentioned experiences were misconceptions from others (n = 7) and judgment/being talked about by others (n = 5). Structural stigma, in terms of being denied opportunities, was mentioned by 1 participant.

### Conceptual model of experiences of people with CHB

**Fig. 2:**
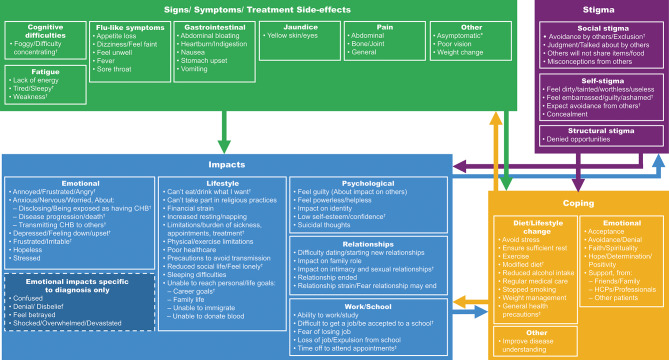
Conceptual model of the experience of patients with CHB. *CHB* chronic hepatitis B, *HBQOL*, Hepatitis B Quality of Life, *HCP*, health care professional. *Asymptomatic is not a sign/symptom or side effect, but people with CHB who are asymptomatic may still experience stigma and other impacts on their daily life. ^†^Concept assessed by the HBQOL. ^‡^Concept reported in the study interviews but not identified in the literature.

The conceptual model of the experiences of people with CHB (Fig. 2) was based on findings from the qualitative literature review and analysis of concept elicitation interviews of people living with CHB in the United Kingdom. Two new concepts were identified during the interviews of this study that were not identified in the literature review. The first was related to time off to attend appointments (n = 6), captured under work/school impact; the second was general health precautions (n = 6), captured under coping strategy.

A total of 59 of 93 symptom/impact/stigma concepts from the conceptual model came up in the first 2 of 3 sets of concept elicitation interviews, with 8 interviews per set that were grouped in sequential order by the date the interviews were conducted (supplemental methods). Of the 7 concepts arising in the last set and the 27 concepts that did not arise in interviews at all, most were symptoms that were experienced heterogeneously among people with CHB, many of whom are asymptomatic. This may reflect that these experiences are individual in nature and not broadly experienced by patients with CHB. The literature review collectively provided qualitative data from many patients from different cultural backgrounds, and thus warrant inclusion in the conceptual model.

### Content validity of the HBQOL

#### Cognitive debriefing of the HBQOL

The review of content validity of the HBQOL [3] included exploration of patient interpretation, understanding and relevance of the questionnaire’s concepts, item wording, instructions, response scales/options, recall period, and evaluation/prioritization of the conceptual framework. Overall, the item wording and response scales/options were understood, and participants were able to use them appropriately to answer the items. Participants had difficulty interpreting words or phrases such as stigmatized (n = 2), anxious (n = 2), influential (n = 3), less productive (n = 2), life expectancy (n = 5), worn out and tired (n = 2), and something serious might be wrong because of your hepatitis B (n = 7).

The results of the cognitive debriefing confirmed the conceptual relevance of the HBQOL items; at least a third of participants endorsed all 31 items as being relevant. Items that assessed worry about disease progression, transmission, worsening health, flare-ups, and life expectancy were endorsed as relevant by ≥ 21 participants (Table 9). However, many participants reported that they did not feel/had never felt isolated from others (n = 12) or stigmatized (n = 10), perhaps a reflection that individuals with CHB do not disclose their HBV status to others. The impact of CHB on self-stigma could be considered a missing item from the measure. Fatigue was commonly experienced by participants, but this concept was not comprehensively assessed by the HBQOL. No other missing concepts were identified.

**Table 9:** Conceptual relevance of the HBQOL items from cognitive debriefing of the HBQOL

	No. reporting (N = 24)
**Relevant to all patients**	
Concern for the development of liver failure because of hepatitis B	24
**Relevant to ≥ 20 patients**	
You might develop liver cancer one day because of your hepatitis B	23
You could transmit hepatitis B to a child	22
Your health might get unexpectedly worse because of hepatitis B	22
I feel sad because of hepatitis B	22
Your hepatitis B may flare up at any time	21
Hepatitis B might affect your life expectancy	21
I feel like something bad might happen because of hepatitis B	21
**Not relevant to ≥ 10 patients**	
I feel like I am less productive because of hepatitis B	15
I feel isolated from others because of hepatitis B	12
I feel stigmatized because of hepatitis	10
Memory loss	10

*HBQOL* Hepatitis B Quality of Life

The HBQOL does not ask responders to remember what they experienced during a specific recall period (e.g., the previous day, week, or month). When asked by the interviewer, most participants said that recall periods of 7 days (n = 21), 2 weeks (n = 19), 24 h (n = 21), and 1 month (n = 19) could be easily used.

### Conceptual framework exploration

Participants were asked to rank HBQOL domains to explore which ones would be most important to people with CHB. Participants ranked anticipation, anxiety, and transmissibility equally as the most important domains (Table 10), and they most frequently ranked disease stigma as the least important.

**Table 10:** HBQOL questionnaire domain ranking results

HBQOL domain	Median rank^*^
Worrying about my health getting worse (anticipation anxiety)	2
The risk of passing my hepatitis B on to somebody else (transmissibility)	2
My mental health (psychological well-being)	3
Limitations to my lifestyle because of hepatitis B (vulnerability)	4
Feeling tired and exhausted (vitality)	5
Other people judging me because of hepatitis B (disease stigma)	6
Other domain suggested by patient: impact on working activities and time off to attend appointments	n/a
Other domain suggested by patient: confidence/ability	n/a

*HBQOL* Hepatitis B Quality of Life, *n/a* not applicable

^*^Patients were asked to rank the HBQOL domains on a scale of 1 (most relevant) to 6 (least relevant)

## Discussion

This study was performed to build a conceptual model of patient experience of CHB and review the content validity of the HBQOL as a measure of CHB symptoms and impacts. The qualitative literature review indicated that people living with CHB experience emotional/psychological impacts and stigma. CHB affects their lifestyle, relationships, and work/school.

In the concept elicitation interviews, fatigue was the most commonly reported, and most participants were worried or anxious about transmitting the virus and about disease progression/death. Others have also noted the importance of chronic fatigue as a primary concern of people with CHB [32]. In this study, the most commonly reported type of stigmatization was self-stigma. Participants’ social functioning was most impacted by the need to take precautions to avoid transmission to others. Combining the results of the qualitative literature review and concept elicitation interviews, a conceptual model of patients’ experiences with CHB was developed to reflect patient experience and help identify patient-relevant PROs.

This study demonstrates the content validity of the HBQOL, including its conceptual relevance, item wording, and response options, in people living with CHB in the United Kingdom [3]. However, the content validity analysis of this questionnaire suggests several potential areas of improvement. Self-stigma, not assessed by the HBQOL, could be measured by a separate self-stigma PRO. A recall period should be added to the HBQOL measure to enable reliable measurement of longitudinal change in patient outcomes. Participants in this study favored the use of a 7-day or 4-week recall period. Finally, rewording of the hard-to-understand words and phrases could improve comprehension of these HBQOL items by participants.

A strength of the interview portion of this study is that the sample size met published recommendations [33]. However, as this study only included patients in the United Kingdom, concept elicitation interviews from different countries/cultures are recommended to confirm the conceptual relevance of the measure. This was partially mitigated by including data of patient experiences with CHB from the literature review of qualitative studies published worldwide in the new conceptual model. The participants in this study appeared to be representative of the population with CHB living in the United Kingdom, which has a high prevalence of people who moved to the United Kingdom with CHB or who acquired it from family members or communities during childhood in the United Kingdom. The high level of education of participants (71% with bachelor’s degree and above) may be higher than for the UK general and CHB-specific populations.

There are some important implications of the current study that should be noted. The present study further supported that the HBQOL assesses concepts that are relevant to patients and showed that some modifications could make it more usable both by patients and in trials in order to measure longitudinal change in HRQoL in patients with HBV. Furthermore, we identified an important concept (self-stigma) that should be measured in CHB trials, which is not assessed by the HBQOL.

## Conclusions

This study demonstrates that CHB has emotional/psychological impacts that affect the HRQoL of patients living with CHB and that their prevalence should not be underestimated. The HBQOL [3] appears to be a conceptually relevant and content-valid PRO measure of the overall HRQoL of patients with CHB. However, some modifications should be considered, such as adding a recall period to evaluate patient outcomes over time and rewording the words and phrases that participants found difficult to understand. In addition, clinical studies using the HBQOL to explore HRQoL should consider including an additional PRO in order to measure self-stigma, which we have identified to be an important concept to patients. For holistic assessment, evaluation of HRQoL aspects that matter most to patients, in addition to functional cure clinical outcomes, should be included in future clinical studies.

### Electronic supplementary material

Below is the link to the electronic supplementary material.

Supplemental methods and tables

## Data Availability

The data sharing policy of Janssen Pharmaceutical Companies of Johnson & Johnson is available at https://www.janssen.com/clinical-trials/transparency. As noted on this site, requests for access to the study data can be submitted through Yale Open Data Access (YODA) Project site at http://yoda.yale.edu.

## Abbreviations

ADL: Activities of daily living
AS: Advanced Subsidiary
BTEC: Business and Technology Education Council
CHB: chronic hepatitis B
CSE: Certificate of Secondary Education
GCSE: General Certificate of Secondary Education
HBQOL: Hepatitis B Quality of Life
HBsAg: hepatitis B surface antigen
HBV: hepatitis B virus
HCP: health care professional
HRQoL: health-related quality of life
PFDD: patient-focused drug development
PRO: patient-reported outcome
STI: sexually transmitted infection
