# Supplementary material for: A conceptual model for chronic hepatitis B and content validity of the Hepatitis B Quality of Life (HBQOL) instrument
Source: J Patient Rep Outcomes. 2024 Mar 4;8:29. doi: 10.1186/s41687-023-00675-8 (PMC10912061; doi:10.1186/s41687-023-00675-8)
Supplement: Supplementary file 1 — Supplemental methods and tables [file 41687_2023_675_MOESM1_ESM.docx]

**Contents**

Supplemental methods………………………………………………………………………………...……..….….2

[Table S1. Results of database searches run March 1, 2021](#_Toc101526351) 4

[Table S2. Details of studies selected for the qualitative literature review](#_Toc101526352) 6

[Table S3. Impact of diagnosis on patients’ lives concept elicitation—summary of responses and example quotes 8](#_Toc101526354)

[Table S4. Coping with CHB concept elicitation—summary of responses and example quotes](#_Toc101526360) 9

References 12

**Supplemental methods**

***Literature search***

Manual searches such as the American Association for the Study of Liver Diseases, Asian Pacific Association for the Study of the Liver, European Association for the Study of the Liver, International Society for Quality of Life Research, International Society for Pharmacoeconomics and Outcomes Research (ISPOR), ISPOR Latin America, ISPOR Europe, and ISPOR Asia Pacific were included.

***Selection of interview participants***

Additional inclusion criteria were sufficient physical, cognitive, reading, and linguistic capacities to allow them to participate in a 60-to-90-minute semi-structured interview. Key criteria for exclusion from the study were having a concurrent diagnosis of cirrhosis, HIV, another type of hepatitis; misusing drugs or alcohol; or any other condition that, in the opinion of the clinician, might impact the results of this study. The clinical characteristics of study participants were captured on case report forms. The study protocol for the concept elicitation and HBQOL content validity interviews was reviewed and approved by the NHS Health Research Authority (REC reference 18/NW/0649, IRAS project ID 247313) on 21 April 2020.

***Interview process***

According to the original protocol, interviews were to be conducted during in-person meetings. However, because of the COVID-19 pandemic and the ensuing lockdown restrictions enacted in the UK, an amendment was submitted and approved to change the interview format from face-to-face to telephone. Thus, all interviews were conducted over the telephone except for one, which used Microsoft Teams.

In preparation for the interview, materials were mailed to study participants with instructions not to view the HBQOL before the interview. Materials provided electronically were sent just prior to the scheduled interview. Interviewers had received training from Collaborative Institutional Training Initiative–certified Clinical Outcome Assessment (COA) interview experts. The same researcher conducted each interview using a semi-structured interview guide divided into the following segments: introduction and consent to participate (~5 minutes); concept elicitation to explore the participant’s experience of living with CHB (15-25 minutes); cognitive debriefing of the HBQOL instrument (30-40 minutes); and discussion to explore the conceptual framework of the HBQOL (10-20 minutes). With participants’ permission, interviews were audio recorded (without collecting any identifying information), given a unique ID, uploaded to a secure server, and transcribed verbatim. To maintain their anonymity, each interview participant was assigned a unique ID (in the order of study recruitment) with a designation of F (female) or M (male). Topics discussed in a non-leading way included symptoms, impact of CHB diagnosis, psychological/emotional well-being, CHB-related stigma, impact on social functioning, impact on relationships, impact on activities of daily living (ADL), impact on work/school, and how study participants coped with CHB. If not discussed, patients were asked specific questions about the relevance of each item to their experiences, their understanding of item wording, their interpretation of each item’s wording, and their views on the appropriateness of the response options. The interviewer also asked the participants if anything important regarding their experiences with CHB was missing from the HBQOL questionnaire. Time permitting, participants were also asked to rank each item within each domain.

***Interview analyses***

Taking a realist semantic approach, the analysts familiarized themselves with the transcripts to generate a broad understanding of the reported patient experience, and then assigned descriptive codes to quotes that described specific signs, symptoms, or impacts. After the full data set had been initially coded, analysts reviewed the codes and merged any that were thought to be describing the same experience. Analysts then combined groups of related codes into domains and compared these domains to assess any relationships between them. For this analysis the interview transcripts were divided into three approximately equal-sized sets, in the sequential order in which they were performed. The transcripts of the concept elicitation segment of the interview from the first set were then compared with those of the second set to identify any new concepts that might have arisen. If no new concepts were identified in the second set, then saturation was considered to have been attained. For any new concept identified by at least four patients in the second set, the transcripts from the third set were reviewed. The code list was developed prior to the interviews, but additional codes were added iteratively during analysis where necessary. Framework analysis is a matrix-based analytic method that facilitates rigorous and transparent data management. A “framework,” or set of main themes and subtopics (i.e., understanding, relevance, and interpretation), were pre-defined, and data items (i.e., each patient interview) were reviewed against this defined list [28].

**Table S1. Results of database searches run March 1, 2021**

| Search no. | Search terms | No. of results |
| --- | --- | --- |
| **Medline and Epub ahead of print, in-process and other non-indexed citations and daily** | | |
| 1 | exp Hepatitis B/ | 59308 |
| 2 | hepatitis B/ or hepatitis b.mp. | 102110 |
| 3 | (hep b or hpv).mp. | 44403 |
| 4 | (chronic hepatitis b virus infection or chronic Hep B or chronic hepatitis b or chronic HBV).mp. | 19992 |
| 5 | exp Hepatitis B Vaccines/ | 9405 |
| 6 | exp hepatitis b virus/ | 27745 |
| 7 | (liver disease and chronic hepatitis b).mp. | 2488 |
| 8 | or/1-7 | 146053 |
| 9 | limit 8 to yr="2019 - 2020" | 13672 |
| 10 | (delta hepatitis or delta infection or hepatitis D or hepatitis delta or HDV).mp. | 4784 |
| 11 | limit 10 to yr="2005 - 2020" | 1828 |
| 12 | qualitative.tw. | 242554 |
| 13 | interview.tw. | 144180 |
| 14 | 12 or 13 | 368818 |
| 15 | 9 or 11 | 15290 |
| 16 | 14 and 15 | 339 |
| 17 | limit 16 to humans | 201 |
| 18 | limit 17 to "all adult (19 plus years)" | 129 |
| 19 | limit 18 to English language | 126 |
| **Embase** | | |
| 1 | exp hepatitis B/ | 103612 |
| 2 | hepatitis B/ or hepatitis b.mp. | 160495 |
| 3 | (hep b or hbv).mp. | 72261 |
| 4 | (chronic hepatitis b virus infection or chronic Hep B or chronic hepatitis b or chronic HBV).mp. | 36963 |
| 5 | exp hepatitis b vaccine/ | 20010 |
| 6 | exp hepatitis b virus/ | 54405 |
| 7 | (liver disease and chronic hepatitis b).mp. | 6836 |
| 8 | or/1-7 | 168741 |
| 9 | limit 8 to yr="2019 - 2020" | 17466 |
| 10 | delta agent hepatitis/ | 3147 |
| 11 | (delta hepatitis or delta infection or hepatitis D or hepatitis delta or HDV).mp. | 6611 |
| 12 | 10 or 11 | 7437 |
| 13 | limit 12 to yr="2005 - 2020" | 3962 |
| 14 | qualitative.tw. | 298909 |
| 15 | interview.tw. | 188471 |
| 16 | 14 or 15 | 462863 |
| 17 | 9 or 13 | 20884 |
| 18 | 16 and 17 | 321 |
| 19 | limit 18 to human | 305 |
| 20 | limit 19 to adult <18 to 64 years> | 217 |
| 21 | limit 20 to english language | 214 |
| **PsycINFO** | | |
| 1 | (hepatitis b or chb or hbv).mp. | 1214 |
| 2 | limit 1 to yr="2019 - 2020" | 79 |
| 3 | (delta hepatitis or delta infection or hepatitis D or hepatitis delta or HDV).mp. | 11 |
| 4 | limit 3 to yr="2005 -2020" | 11 |
| 5 | 2 or 4 | 90 |
| 6 | limit 5 to ("0700 interview" or "0750 focus group" or 1600 qualitative study) | 16 |

**Table S2. Details of studies selected for the qualitative literature review**

| **Study name** | **Data collection method** | **No. of study participants** | **Country** |
| --- | --- | --- | --- |
| Adjei et al, 2017[34] | In-depth interviews | 14 | Ghana |
| Adjei et al, 2019,[35, 36] 2020[37] | Semi-structured interviews | 18 | Ghana |
| Allard et al, 2018[38] | Semi-structured interviews | 19 | Australia |
| Anderson et al, 2016[39] | Face-to-face interviews and questionnaires | 42 | Australia |
| Chabrol et al, 2019[40] | In-depth interviews | 8 | Cameroon |
| Franklin et al, 2018[41] | Quantitative survey and focus group | 79 | Zambia |
| Giles-Vernick et al, 2016[42] | Semi-structured interviews | 28 | Burkina Faso |
| Hajarizadeh et al, 2014[43] | Semi-structured interviews | 10 | Australia |
| Hassani et al, 2017[44]* | Semi-structured interviews | 32 | Iran |
| Heaney et al, 2012[45] | In-depth interviews | 127 | European Union |
| Jang et al, 2018[46] | Face-to-face interviews | 11 | South Korea |
| Lee et al, 2010[47] | Semi-structured interviews | 9 | South Korea |
| Lee et al, 2011[48] | Semi-structured interviews | 12 | United States |
| Le Gautier et al, 2019,[49]^†^  2021[50] | Semi-structured interviews | 37 | Australia |
| Li et al, 2019[51] | Semi-structured interviews | 4 | China |
| Lin et al, 2019[52] | Semi-structured interviews | 47 | Taiwan |
| Mude et al, 2019,[53] 2020[54] | Face-to-face interviews | 15 | Australia |
| Ng et al, 2013[55] | Focus groups | 44 | Malaysia |
| Nishimura et al, 2012[18] | Survey with open-ended components | 829 | United States |
| Oka et al, 2017[56] | Semi-structured interviews | 107 | Japan |
| Richmond et al, 2017[57] | Semi-structured interviews | 19 | Australia |
| Sievert et al, 2018[58] | Semi-structured interviews | 19 | Australia |
| Spiegel et al, 2007[3] | Semi-structured interviews and focus group | 59 | United States |
| Taheri Ezbarami et al, 2017[59]* | Semi-structured interviews | 27 | Iran |
| **Study name** | **Data collection method** | **No. of study participants** | **Country** |
| Tan et al, 2005[60] | Focus groups | 39 | Singapore |
| Teston et al, 2013[61] | Semi-structured interviews | 12 | Brazil |
| Tha et al, 2014[62] | Focus groups | 19 | Malaysia |
| Valizadeh et al, 2016,[63]^‡^ 2017,[64] 2019[65] | Unstructured, in-depth interviews | 18 | Iran |
| Wallace et al, 2007[66] | Semi-structured interviews | 20 | Australia |
| Wallace et al, 2011[67] | Semi-structured interviews | 20 | Australia |
| Wallace et al, 2015[68] | Semi-structured interviews | Not reported | China |
| Wallace et al, 2015[69] | Semi-structured interviews | 46 | Not reported |
| Wallace et al, 2017[70] | Semi-structured interviews | 41 | China |
| Yao et al, 2015[71] | Social media review In-depth interviews | 1838 13 | China |
| Zheng et al, 2019,[72] 2020[73] | Semi-structured interviews and brief questionnaire | 32 | China |
| Zhu et al, 2019[74] | Semi-structured interviews | 94 | China |

*Although Hassani et al, 2017,[44] and Taheri Ezbarami et al, 2017,[59] report findings from the same overall study, these publications have been extracted as though they were distinct studies because they have different objectives and numbers of participants.

^†^Le Gautier et al, 2019, includes findings only from a subgroup of Vietnamese participants.

^‡^Valizadeh et al, 2016, reports data from only n=15/18 participants.

## Table S3. Impact of diagnosis on patients’ lives concept elicitation—summary of responses and example quotes

| Impact of diagnosis on patients’ lives | No. reporting (N=24) | Example quotes |
| --- | --- | --- |
| Shocked/ Overwhelmed/ Devastated | 6 | “It came as a shock to me because I was never aware of it, and I attended the hospital for -- because I suffer from diabetes. I did [redacted] and the ambulance picked me up from [redacted] and they brought me there. So I had a blood test and I was treated there, then I went home. Then I think couple of days after that, I got a phone call and I was informed that I got hep B. And I was like, oh, okay. And at first, I didn't know nothing about it. Then I felt like, okay, let me just explore this and see what is here. And I went to Google it and I find out what could this be? What is it all about? And the effects and everything else. I'm like, wow, okay? How did I get it? I wasn't too sure how I got it, you know.”  “Well, I was shocked at first to find this out. I was a bit upset and devastated that I was so young and yet I had to live with this for the rest of my life. Now I’m a bit numb about it. I’m like, ‘Oh, it is what it is, just deal with it.’” |
| Confusion | 5 | “The questions, why? How did it happen and why couldn't I prevent it? I don't know. I mean, I've been thinking about why, why, why, where, where, where? How did it happen? When did it happen?” |
| Denial/ disbelief | 3 | “To start with, when I first got diagnosed, it was pretty scary. I wasn’t quite sure what the diagnosis meant, and I went into denial. So I kept trying to change my appointment on this computerized system so I did not have to go see the doctor. But in the end, they got wise to me, and they said no, you’ve got to come and see us.” |
| Feel betrayed | 2 | “The disappointment that maybe it wasn’t discovered earlier, and also some kind of disappointment that maybe I wasn’t vaccinated, I guess. I mean, to prevent it in the first instance. Yeah, yeah, so it was more surprise and disappointment and […] healthcare of the system where I grew up.” |

## Table S4. Coping with CHB concept elicitation—summary of responses and example quotes

| Coping technique | No. reporting (N=24) | Example quotes |
| --- | --- | --- |
| Acceptance | 14 | “But at the moment since, I know it’s part of me. And after all, everybody will die one day. So emotionally it affected me, but as I’m speaking now, I don’t think about it. I don’t think about it emotionally.  “I have to be at peace with it and talking about my experiences. It’s not like I have done something to contract it. I was not cautious or leading that type of life when I got it. It was just the cards dealt in this case.”  “For me, it's fine. I don't really think about it that much. The people who are closest to me that know about it, I've asked them to get tested to make sure that I haven't impacted anybody else. And I've made my peace with it. So, it's fine.” |
| Regular medical care | 10 | “And since the virus went down, because when I started for the first month, I went back to the hospital and they told me the viral load was 68 million but it has reduced. I remember for about six months it took me to reduce for something like that. So I was very happy. And from 68 million to 500 this means the medication is working. And now I remember I know it’s below 20, which they told me since it’s below 20, it’s fine. But I should keep taking the medication so that they are not able to re-multiply. So since I got to know this, I’m happy because I think the medication is working.”  “Generally, yeah, it’s fine. Don’t have any major issues living with it. I just take my medication regularly on a daily basis and to look for the appointments and everything seems well so it seems okay now. I have regular biopsies as well.” |
| Support from family/ friends | 7 | “I'm very lucky. My partner knows and her friends, they -- some of them realize, the very, very close ones I trust. I can confide to. They know what's happened.”  “I think I’m quite lucky because I have a family and they’ve been very supportive, and yeah, I think you leave to live with it and, like, never goes away, it’s always in the back of your mind.” |
| Improve disease understanding | 7 | “And at that point in time, my level of being ashamed then go down a little bit because I felt like, rather than being ashamed, I should better be some kind of preacher who talk to people more about things like this to pay more attention because there's other millions of people back home in [redacted] who can actually pay attention to that. I spoke to my cousins about it, spoke to sister, spoke to my brothers. But I told, "Look, keep preaching it. If people can get themself test, please family around.” And then out from that, I kind of find I got a lot of feedback, connections to different people that have been in touch with that other 10 people who also confirm that they've got it already out of hundred people that they are reaching out to. And I'm still reaching out now, telling people, "Please keep checking yourself.” Because they said, "The earlier you discover you got it, then the more you can start some kind of treatment and start helping it. Yeah. That kind of bring my level of being ashamed down.”    “I don't know. Sometimes, at the beginning, I was worried because I don't know what it is. I was young as well. Not too many information. But with the time and I start to see the doctors. In the time I tried to read more. And I start to settle down and be more confident, and be more okay with me. So, it's not such a bad thing.” |
| General health precautions | 6 | “I tried to have a healthy life as much as I can.”  “I do take care of myself generally, especially past the 30 birthday mark, so yes, I would say I’ve got it quite under control.” |
| Reduced alcohol intake | 6 | “Alcohol intake, so I don’t drink that much, but I work in [redacted] which means you’re sometimes mingling with your teams, and clients which -- sometimes excessive drinking, but, so, sometimes I just have to be mindful of the alcohol intake, and beyond that I don’t feel limited in anyway.”  “Yeah. I suppose my immediate reaction was to just stop drinking alcohol altogether. Because even the risk to liver damage was very, very small, I just said, well, I'm just not going to drink anything at all. And I don't know how long that lasted for, a few months. And, then, I said, it's safe for me to have one or two drinks. And in the long term, I've just cut down on how much I drink as a result of it.” |
| Support from HCPs/ Professionals | 6 | “And then, thankfully, I met with, I think it was, Dr. [redacted] at that time. He walked me through what it is I was going through, and he talked to me […] give me a lot of materials and things, so I begin to read because I'm the kind of person who also loves to read, so I know what I'm going through.”    “Yeah, absolutely. And I think the [redacted], the [redacted], and also the team, the research team at [redacted] just really lovely people. You know, very down to earth, obviously not judgmental at all. So it’s been a real catalyst for change. So it’s really, really good.” |
| Modified diet | 5 | “Also I'm very precautious not taking too much fatty foods and stuff. It's already even I think […], not taking too much sweets and other stuff. I don't like sweet things like we use in celebration and other stuff.” |
| Hope/ determination/ positivity | 4 | “I’m the type of person that doesn’t let things affect me, to be honest. I said, “You know what? Life’s too short, just live it the best way you can even if it’s going to be a bit shorter, but at least you won’t have any regrets and you’ll feel happy that you haven’t stopped yourself from doing things.” |
| Exercise | 3 | “Yeah, it’s been quite transformational. So I think I was just fumbling along like you normally do, in your sort of mid-40s, and getting a bit of weight on, and sort of relaxing a bit. And then having had that diagnosis, deciding to sort of completely transform my approach to life and living, and significantly improve my quality of life as a result. So I’ve been sort of an outdoor person and doing lots of exercise, and so I went back to all of that. And really stopped drinking almost all together, although I do have a little bit, few more glasses of wine these days. And started cycling and working out and walking out and all of this sort, and have lost quite a lot of weight. And with the recent lockdown, I’ve now just started running. So now I found myself in 30 years [INDISCERNIBLE] fixed state, and doing a triathlon. So I get up in the morning, I do a workout, and then I go for a run. And then I do a bike ride. So just to get through the lockdown, and that’s all before 9:00. And I’ve gotten myself a Fitbit and things like that. So, yeah, it’s just a much better quality of life. I sleep better, and I’m much trimmer. I feel better. Yes, it’s been a real wakeup call, a real catalyst for me to sort myself out. So now I’m nearly [redacted], I feel fitter than I’ve ever been all my life. Shocking isn’t it?” |
| Avoidance/ denial | 2 | Interviewer: Can you tell me how Hep B has affected you emotionally?  “I'm trying to avoid -- to avoid that. Every now and then, the reminder I get from the medical follow-up, the information that I share with my GP to partially trigger or remind me of the fact that it is -- it is serious and, yes, I can't -- I can't avoid thinking about the relative stages. But I don't know much about because so far I don't have any sign.” |
| Avoid stress | 1 | “I try not to think too much, you know?” |
| Ensure sufficient rest | 1 | “I guess one particular thing is that I mean I can’t really stay late like midnight or I mean I don’t know whether it’s due to -- it probably is my -- it’s health way anyway, so I try to stay before midnight.” |
| Stopped smoking | 1 | “I have the same activity. Not affect me too much, to be honest. Because I try to be healthy and eat healthy food. I'm not drinking, I'm not smoking. I don't know if they affect me or not.” |
| Faith/ spirituality | 1 | “Yeah. I took the therapy, the professional therapy sessions. Yeah … I found it helpful and, also, I think other thing was helpful was my religious side of it.” |

CHB, chronic hepatitis B.

**References**

1. World Health Organization (2020) Hepatitis B. <https://www.who.int/en/news-room/fact-sheets/detail/hepatitis-b>. Accessed June 23, 2022,.

2. European Association for the Study of the Liver (2017) EASL 2017 Clinical Practice Guidelines on the management of hepatitis B virus infection. J Hepatol 67(2):370-398. doi: 10.1016/j.jhep.2017.03.021.

3. Spiegel BM, Bolus R, Han S, Tong M, Esrailian E, Talley J, et al. (2007) Development and validation of a disease-targeted quality of life instrument in chronic hepatitis B: the hepatitis B quality of life instrument, version 1.0. Hepatology 46(1):113-121. doi: 10.1002/hep.21692.

4. Tu T, Block JM, Wang S, Cohen C, Douglas MW (2020) The lived experience of chronic hepatitis B: a broader view of its impacts and why we need a cure. Viruses 12(5):515. doi: 10.3390/v12050515.

5. Freeland C, Racho R, Kamischke M, Moraras K, Wang E, Cohen C, et al. (2021) Health-related quality of life for adults living with hepatitis B in the United States: a qualitative assessment. J Patient Rep Outcomes 5(1):121. doi: 10.1186/s41687-021-00398-8.

6. Sarin SK, Kumar M, Lau GK, Abbas Z, Chan HL, Chen CJ, et al. (2016) Asian-Pacific clinical practice guidelines on the management of hepatitis B: a 2015 update. Hepatol Int 10(1):1-98. doi: 10.1007/s12072-015-9675-4.

7. FDA (2021) FDA-led patient-focused drug development (PFDD) public meetings. <https://www.fda.gov/industry/prescription-drug-user-fee-amendments/fda-led-patient-focused-drug-development-pfdd-public-meetings>. Accessed June 23, 2022,.

8. Hunter NL, O'Callaghan KM, Califf RM (2015) Engaging patients across the spectrum of medical product development: view from the US Food and Drug Administration. JAMA 314(23):2499-2500. doi: 10.1001/jama.2015.15818.

9. FDA (2021) CDER Patient-Focused Drug Development. <https://www.fda.gov/drugs/development-approval-process-drugs/cder-patient-focused-drug-development>. Accessed June 23, 2022,.

10. European Medicines Agency (2021) ICH reflection paper - proposed ICH guideline work to advance Patient Focused Drug Development (PFDD). <https://www.ema.europa.eu/en/documents/scientific-guideline/ich-reflection-paper-proposed-ich-guideline-work-advance-patient-focused-drug-development-pfdd_en.pdf>. Accessed.

11. European Medicines Agency (2020) ICH reflection paper on proposed ICH guideline work to advance patient focused drug development <https://www.ema.europa.eu/en/documents/scientific-guideline/ich-reflection-paper-proposed-ich-guideline-work-advance-patient-focused-drug-development_en.pdf>. Accessed June 23, 2022.

12. Food & Drug Administration (2017) Plan for issuance of patient‐focused drug development guidance under 21st Century Cures Act Title III Section 3002. <https://www.fda.gov/files/about%20fda/published/Plan-for-Issuance-of-Patient%E2%80%90Focused-Drug-Development-Guidance.pdf>. Accessed.

13. National Health Council (2019) The National Health Council rubric to capture the patient voice: a guide to incorporating the patient voice into the health ecosystem. <https://nationalhealthcouncil.org/additional-resources/patient-engagement-rubric/>. Accessed June 23, 2022.

14. European Medicines Agency (2005) Reflection paper on the regulatory guidance for the use of health-related quality of life (HRQL) measures in the evaluation of medicinal products. <https://www.ema.europa.eu/en/documents/scientific-guideline/reflection-paper-regulatory-guidance-use-healthrelated-quality-life-hrql-measures-evaluation_en.pdf>. Accessed June 23, 2022.

15. Food and Drug Administration (2009) Patient-reported outcome measures: use in medical product development to support labeling claims: guidance for industry. <https://www.fda.gov/regulatory-information/search-fda-guidance-documents/patient-reported-outcome-measures-use-medical-product-development-support-labeling-claims>. Accessed June 23, 2022.

16. Powers JH, 3rd, Howard K, Saretsky T, Clifford S, Hoffmann S, Llorens L, et al. (2016) Patient-reported outcome assessments as endpoints in studies in infectious diseases. Clin Infect Dis 63(Suppl 2):S52-56. doi: 10.1093/cid/ciw317.

17. Verma M (2020) Patient reported outcomes as emerging biomarkers in chronic liver disease research. J Hepatol 72(6):1215-1216. doi: 10.1016/j.jhep.2020.02.010.

18. Nishimura A, Shiono P, Stier D, Shallow S, Sanchez M, Huang S (2012) Knowledge of hepatitis B risk factors and prevention practices among individuals chronically infected with hepatitis B in San Francisco, California. J Community Health 37(1):153-158. doi: 10.1007/s10900-011-9430-2.

19. Patrick DL, Burke LB, Gwaltney CJ, Leidy NK, Martin ML, Molsen E, et al. (2011) Content validity--establishing and reporting the evidence in newly developed patient-reported outcomes (PRO) instruments for medical product evaluation: ISPOR PRO Good Research Practices Task Force report: part 1--assessing respondent understanding. Value Health 14(8):978-988. doi: 10.1016/j.jval.2011.06.013.

20. Patrick DL, Burke LB, Gwaltney CJ, Leidy NK, Martin ML, Molsen E, et al. (2011) Content validity--establishing and reporting the evidence in newly developed patient-reported outcomes (PRO) instruments for medical product evaluation: ISPOR PRO Good Research Practices Task Force report: part 2--assessing respondent understanding. Value Health 14(8):978-988. doi: 10.1016/j.jval.2011.06.013.

21. Terwee CB, Prinsen CAC, Chiarotto A, Westerman MJ, Patrick DL, Alonso J, et al. (2018) COSMIN methodology for evaluating the content validity of patient-reported outcome measures: a Delphi study. Qual Life Res 27(5):1159-1170. doi: 10.1007/s11136-018-1829-0.

22. Zumbo DB, Chan EKHe (2014) Validity and validation in social, behavioral, and health sciences. 1 ed. Springer International Publishing Switzerland,

23. Lok AS, Zoulim F, Dusheiko G, Ghany MG (2017) Hepatitis B cure: from discovery to regulatory approval. Hepatology 66(4):1296-1313. doi: 10.1002/hep.29323.

24. Trigg A, Andersson FL, Aldhouse NVJ, Bliwise DL, Kitchen H (2017) Patients' lived experiences of nocturia: a qualitative study of the evening, the night, and the next day. Patient 10(6):711-718. doi: 10.1007/s40271-017-0241-0.

25. Critical Appraisal Skills Programme (2021) CASP Systematic Review Checklist. <https://casp-uk.net/casp-tools-checklists/>. Accessed June 23, 2022,.

26. Guest G, Bunce A, Johnson L (2006) How many interviews are enough?: an experiment with data saturation and variability. Field Methods 18(1):59-82. doi: 10.1177/1525822x05279903.

27. Braun B (2006) Phonetics and phonology of thematic contrast in German. Lang Speech 49(Pt 4):451-493. doi: 10.1177/00238309060490040201.

28. Ritchie J, Spencer L, O'Connor W (2003) Carrying out qualitative analysis. Qualitative Research Practice: A Guide for Social Science Students and Researchers London. SAGE, pp 219-262.

29. World Health Organization (2001) The World Health Report 2001: mental health: new understanding, new hope. <https://apps.who.int/iris/handle/10665/42390>. Accessed June 23, 2022.

30. Boyd JE, Adler EP, Otilingam PG, Peters T (2014) Internalized Stigma of Mental Illness (ISMI) scale: a multinational review. Compr Psychiatry 55(1):221-231. doi: 10.1016/j.comppsych.2013.06.005.

31. Sheehan L, Nieweglowski K, Corrigan PW (2017) Structures and types of stigma. In: Gaebel W, Roessler W, Sartorius N (eds) The Stigma of Mental Illness - End of the Story? Springer International Publishing, Cham, Switzerland, pp 43-66.

32. Hepatitis B Foundation (2020) The voice of the patient report: living with chronic hepatitis B. Journal (Issue). doi:

33. Turner-Bowker DM, Lamoureux RE, Stokes J, Litcher-Kelly L, Galipeau N, Yaworsky A, et al. (2018) Informing a priori sample size estimation in qualitative concept elicitation interview studies for clinical outcome assessment instrument development. Value Health 21(7):839-842. doi: 10.1016/j.jval.2017.11.014.

34. Adjei CA, Naab F, Donkor ES (2017) Beyond the diagnosis: a qualitative exploration of the experiences of persons with hepatitis B in the Accra Metropolis, Ghana. BMJ Open 7(11):e017665. doi: 10.1136/bmjopen-2017-017665.

35. Adjei CA, Stutterheim SE, Naab F, Ruiter RAC (2019) Chronic Hepatitis B stigma in Ghana: a qualitative study with patients and providers. BMJ Open 9(6):e025503. doi: 10.1136/bmjopen-2018-025503.

36. Adjei CA, Stutterheim SE, Naab F, Ruiter RAC (2019) Barriers to chronic Hepatitis B treatment and care in Ghana: a qualitative study with people with Hepatitis B and healthcare providers. PLoS One 14(12):e0225830. doi: 10.1371/journal.pone.0225830.

37. Adjei CA, Stutterheim SE, Naab F, Ruiter RAC (2020) "To die is better than to tell": reasons for and against disclosure of chronic hepatitis B status in Ghana. BMC Public Health 20(1):663. doi: 10.1186/s12889-020-08811-5.

38. Allard N, Emery J, Cowie B, Furler J (2018) Knowing and telling: how African-Australians living with chronic hepatitis B understand hepatocellular carcinoma risk and surveillance. Aust J Prim Health 24(2):141-148. doi: 10.1071/PY17099.

39. Anderson E, Ellard J, Wallace J (2016) Torres Strait Islanders' understandings of chronic hepatitis B and attitudes to treatment. Aust J Prim Health 22(4):316-319. doi: 10.1071/PY14130.

40. Chabrol F, Noah Noah D, Tchoumi EP, Vidal L, Kuaban C, Carrieri MP, et al. (2019) Screening, diagnosis and care cascade for viral hepatitis B and C in Yaounde, Cameroon: a qualitative study of patients and health providers coping with uncertainty and unbearable costs. BMJ Open 9(3):e025415. doi: 10.1136/bmjopen-2018-025415.

41. Franklin S, Mouliom A, Sinkala E, Kanunga A, Helova A, Dionne-Odom J, et al. (2018) Hepatitis B virus contact disclosure and testing in Lusaka, Zambia: a mixed-methods study. BMJ Open 8(9):e022522. doi: 10.1136/bmjopen-2018-022522.

42. Giles-Vernick T, Hejoaka F, Sanou A, Shimakawa Y, Bamba I, Traore A (2016) Barriers to linkage to care for Hepatitis B virus infection: a qualitative analysis in Burkina Faso, West Africa. Am J Trop Med Hyg 95(6):1368-1375. doi: 10.4269/ajtmh.16-0398.

43. Hajarizadeh B, Wallace J, Ngo N, Richmond J (2014) Hepatitis B Patient and Clinical Practice Survey. <https://www.semanticscholar.org/paper/Hepatitis-B-patient-and-clinical-practice-survey.-Hajarizadeh-Wallace/4c2e9da49e8bc67b0a9d1ed179ea965898bf3f97>. Accessed June 23, 2022.

44. Hassani P, Taheri Ezbarami Z, Zagheri Tafreshi M, Alavi Majd H (2017) A qualitative study on marital challenges of chronic Hepatitis B patients. Iran Red Crescent Med J 19(8):e55577. doi: 10.5812/ircmj.55577.

45. Heaney G, Fletcher S, Murtezaoglu A (2012) 986 Evaluating the patient-physician relationship in chronic hepatits B: results from a qualitative European patient survey. J Hepatol 56(Suppl 2):S385. doi:

46. Jang Y, Boo S, Yoo H (2018) Hepatitis B virus infection: fatigue-associated illness experiences among Koreans Gastroenterol Nurs 41(5):388-395. doi: 10.1097/SGA.0000000000000335.

47. Lee H, Yang JH, Cho MO, Fawcett J (2010) Complexity and uncertainty of living with an invisible virus of hepatitis B in Korea. J Cancer Educ 25(3):337-342. doi: 10.1007/s13187-010-0047-4.

48. Lee H, Hann HW, Yang JH, Fawcett J (2011) Recognition and management of HBV infection in a social context. J Cancer Educ 26(3):516-521. doi: 10.1007/s13187-011-0203-5.

49. Le Gautier R, Wallace J, J AR, Pitts M (2019) The role of explanatory models of chronic hepatitis B on illness experience: a qualitative study of Vietnamese participants in Australia. Ethn Health:1-17. doi: 10.1080/13557858.2019.1612519.

50. Le Gautier R, Wallace J, Richmond JA, Pitts M (2021) The personal and social impact of chronic hepatitis B: A qualitative study of Vietnamese and Chinese participants in Australia. Health Soc Care Community 29(5):1420-1428. doi: 10.1111/hsc.13197.

51. Li T, Su S, Zhao Y, Deng R, Fan M, Wang R, et al. (2019) Barriers to the prevention and control of Hepatitis B and Hepatitis C in the community of Southwestern China: a qualitative research. Int J Environ Res Public Health 16(2):231. doi: 10.3390/ijerph16020231.

52. Lin WS, Lee TT, Yang YH, Mills ME (2019) Environmental factors affecting self-management of chronic hepatitis B from the patients' perspective. J Clin Nurs 28(21-22):4128-4138. doi: 10.1111/jocn.14973.

53. Mude W, Fisher C, Richmond J, Wallace J, Le Gautier R (2019) A qualitative investigation of barriers, support-seeking and coping among South Sudanese people with chronic hepatitis B in Australia. Australian Journal of Primary Health 25(3):264-274. doi: <https://doi.org/10.1071/PY19015>.

54. Mude WW, Fisher CM, Richmond J, Gautier RL, Wallace J (2020) Social impacts of living with chronic hepatitis B in South Sudanese community in Australia. Ethn Health:1-13. doi: 10.1080/13557858.2020.1782849.

55. Ng CJ, Low WY, Wong LP, Sudin MR, Mohamed R (2013) Uncovering the experiences and needs of patients with chronic hepatitis B infection at diagnosis: a qualitative study. Asia Pac J Public Health 25(1):32-40. doi: 10.1177/1010539511413258.

56. Oka T, Enoki H, Tokimoto Y, Kawanishi T, Minami M, Okuizumi T, et al. (2017) Employment-related difficulties and distressed living condition in patients with hepatitis B virus: A qualitative and quantitative study. BMC Public Health 17(1):568. doi: 10.1186/s12889-017-4416-3.

57. Richmond J, Smith E, Wallace J, Duncan D, Lucke J (2017) Hepatitis B testing and diagnosis experiences of patients and primary care professionals in Australia. Aust Fam Physician 46(7):513-519. doi:

58. Sievert K, O'Neill P, Koh Y, Lee JH, Dev A, Le S (2018) Barriers to accessing testing and treatment for chronic Hepatitis B in Afghan, Rohingyan, and South Sudanese populations in Australia. J Immigr Minor Health 20(1):140-146. doi: 10.1007/s10903-017-0546-z.

59. Taheri Ezbarami Z, Hassani P, Zagheri Tafreshi M, Alavi Majd H (2017) A qualitative study on individual experiences of chronic hepatitis B patients. Nurs Open 4(4):310-318. doi: 10.1002/nop2.100.

60. Tan NC, Cheah SL, Teo EK (2005) A qualitative study of health-seeking behavior of Hepatitis B carriers. Singapore Med J 46(1):6-10. doi:

61. Teston EF, Silva RL, Marcon SS (2013) [Living with hepatitis: impact on the daily life of infected subjects]. Rev Esc Enferm USP 47(4):860-868. doi: 10.1590/S0080-623420130000400013.

62. Tha KK, Ng CJ, Tong WT, Kadar SA, Low WY, Mohamed R (2014) Why do patients with chronic hepatitis B use complementaryand alternative medicine? Hepatol Int 8:S90. doi:

63. Valizadeh L, Zamanzadeh V, Negarandeh R, Zamani F, Hamidia A, Zabihi A (2016) Psychological reactions among patients with chronic hepatitis B: a qualitative study. J Caring Sci 5(1):57-66. doi: 10.15171/jcs.2016.006.

64. Valizadeh L, Zamanzadeh V, Bayani M, Zabihi A (2017) The social stigma experience in patients with hepatitis B infection: a qualitative study. Gastroenterol Nurs 40(2):143-150. doi: 10.1097/SGA.0000000000000223.

65. Valizadeh L, Zamanzadeh V, Zabihi A, Negarandeh R, Jafarian Amiri SR (2019) Qualitative study on the experiences of hepatitis B carriers in coping with the disease. Jpn J Nurs Sci 16(2):194-201. doi: 10.1111/jjns.12229.

66. Wallace J, McNally S, Richmond J (2007) National hepatitis B needs assessment report - Australia. <https://apo.org.au/sites/default/files/resource-files/2007-02/apo-nid1730.pdf>. Accessed June 23, 2022.

67. Wallace J, McNally S, Richmond J, Hajarizadeh B, Pitts M (2011) Managing chronic hepatitis B: A qualitative study exploring the perspectives of people living with chronic hepatitis B in Australia. BMC Res Notes 4:45. doi: 10.1186/1756-0500-4-45.

68. Wallace J, Pitts M, Liu C, Lin V, Wei L, Hajarizadeh B, et al. (2015) Viral hepatitis control in China a systematic identification of the barriers to clinical treatment. Hepatol Int 9(Suppl 1):S20. doi:

69. Wallace J, Pitts M, Liu C, Lin V, Wei L, Hajarizadeh B, et al. (2015) Needs assessment of people with viral hepatitis – China. <https://www.latrobe.edu.au/__data/assets/pdf_file/0006/649302/China-Needs-Assessment-English.pdf>. Accessed June 12, 2023.

70. Wallace J, Pitts M, Liu C, Lin V, Hajarizadeh B, Richmond J, et al. (2017) More than a virus: a qualitative study of the social implications of hepatitis B infection in China. Int J Equity Health 16(1):137. doi: 10.1186/s12939-017-0637-4.

71. Yao T, Zheng Q, Fan X (2015) The impact of online social support on patients' quality of life and the moderating role of social exlusion. J Serv Res 18(3):369-383. doi: <https://doi.org/10.1177/1094670515583271>.

72. Zheng YJ, Zhu l, Zhang L, Patrick DL, Wang HM (2019) (3041) Work-health-personal life conflict of new chronic hepatitis B patients in China: a qualitative study. Qual Life Res 28(Suppl 1):S161. doi: <https://doi.org/10.1007/s11136-019-02257-y>.

73. Zheng Y, Zhu L, Patrick D, Li Y, Xu F, Zhang L, et al. (2020) Work-health-personal life conflicts in naive patients with chronic hepatitis B receiving initial treatment in China: a qualitative study. BMJ Open 10(9):e035688. doi: 10.1136/bmjopen-2019-035688.

74. Zhu L, Kong J, Zheng Y, Song M, Cheng X, Zhang L, et al. (2019) Development and initial validation of the chronic hepatitis B quality of life instrument (CHBQOL) among Chinese patients. Qual Life Res 28(11):3071-3081. doi: 10.1007/s11136-019-02240-7.
